# Syndrome d′Apple-Peel avec nécrose intestinale

**Published:** 2012-11-23

**Authors:** Khalid Khattala, Youssef Bouabdallah

**Affiliations:** 1 Service de chirurgie pédiatrique CHU Hassan II, Fès, Maroc

**Keywords:** Syndrome d'Apple-Peel, nècrose intestinale, intestin grêle, atrèsie intestinale, Apple-Peel syndrom, intestinal necrosis, small intestin, intestinal atresia

## Images en médecine

Le syndrome d'Apple-Peel est une forme rare d'atrésie du grêle, dû à une occlusion de l'artère mésentérique supérieure, se manifeste par un enroulement du grêle autour de l'axe vasculaire en colimaçon ou en queue de cochon, la nécrose du grêle associée est encore plus rare. Le traitement repose sur le rétablissement de la continuité qui se fait en un ou en deux temps, sous couvert d'une alimentation parentérale prolongée. Nous rapportons le cas d'un nouveau né de sexe masculin, admis à 8 heures de vie pour une occlusion néonatale avec abdomen plat, l'examen a trouvé un nouveau né en bon état général avec examen clinique normal, bilan biologique normal, une radiographie thoraco abdominale a montré quelques niveaux hydro-aériques de type grêliques, l'exploration chirurgicale a mis en évidence une atrésie grêlique type V, appelée également syndrome d'Apple-Peel avec nécrose étendu du grêle. Vu la non disponibilité de l'alimentation parentérale, le bébé est décédé deux jours en postopératoire.

**Figure 1:**
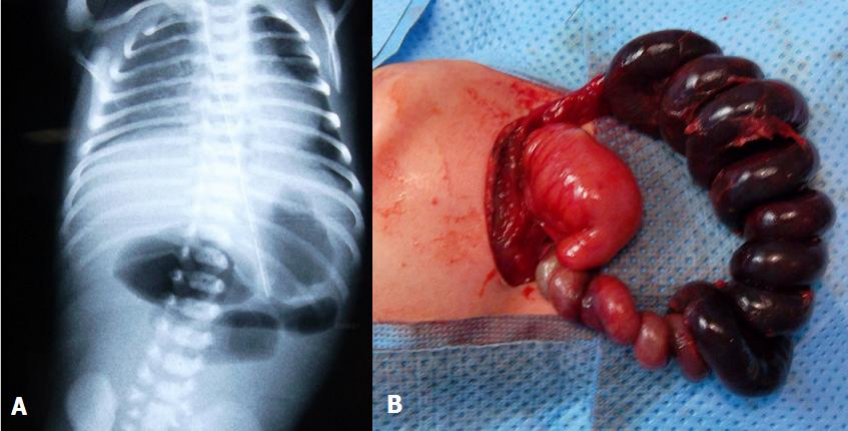
(A) Radiographie thoraco- abdominale sans préparation montrant des niveaux hydro-aériques de type grêlique; (B): aspect per-opératoire objectivant une atrésie grêlique en queue de cochon avec nécrose de la presque totalité des anses intestinales

